# Acceptability of mass drug administration for lymphatic filariasis in Baglung Municipality of Nepal: A mixed-method study

**DOI:** 10.1371/journal.pgph.0004910

**Published:** 2025-07-21

**Authors:** Muskan Pudasainee, Damaru Prasad Paneru, Baburam Acharya, Prabin Sharma, Anil Khanal, Bibash Rana, Shiksha Adhikari, Chiranjivi Adhikari

**Affiliations:** 1 Pokhara University, School of Health and Allied Sciences, Pokhara, Kaski, Nepal; 2 Health Office Baglung, Baglung, Gandaki, Nepal; 3 Health Office Parbat, Parbat, Gandaki, Nepal; PLOS: Public Library of Science, UNITED STATES OF AMERICA

## Abstract

**Introduction:**

Lymphatic filariasis (LF), a neglected tropical disease, remains a significant public health challenge in endemic regions including Baglung Municipality of Nepal. This study investigates the acceptability of mass drug administration (MDA) for LF in Baglung Municipality of Nepal.

**Methodology:**

A cross-sectional, mixed-method study was conducted in Baglung Municipality among 272 community people. Three Focus Group Discussions (FGD) were conducted among 25 commmunity members. Quantitative data were collected using pretested structured questionnaires and FGDs were conducted using structured guideline. Probability proportional to size sampling was done to select desired number of participants. EpiData 3.1 was used for data entry and IBM SPSS 22 was employed for quantitative data analysis. Descriptive statistics and logistic regression was applied. Similarly, thematic analysis was done for qualitative data analysis.

**Results:**

The study revealed that 84.2% were aware of MDA with 48.5% of participants having good knowledge about LF. About 59.3% and 45.2% had a positive attitude towards MDA and LF respectively. More than four fifth (80.5%) of the participants had accepted the MDA campaign with 91.5% having received MDA medication. Significant factors for MDA acceptability included educational status, attitude towards MDA, fear of side effects, and medication received. Qualitative data highlighted the strong influence of effective communication by health workers on MDA acceptance.

**Conclusion:**

Knowledge on LF was found low among the participants with lesser proportion having positive attitude towards LF; highlighting the need for an increase in knowledge and attitude towards LF. The study shows the high level of acceptability of MDA; primarily influenced by the effective communication by health workers and the public trust in health services. Health workers led community-based strategies and programs are recommended for the high acceptance of MDA and the development of a positive attitude for better compliance.

## Introduction

Lymphatic filariasis (LF) is a neglected parasitic disease that is considered to be the second leading cause of permanent and long-term disabilities in 2021 [1–3]. Infection occurs when filarial parasites are transmitted to humans through mosquitoes [4] and it can seriously affect the lives of people [5]. The burden of LF is most prominent among low-income populations in rural areas, where access to health care is limited [6].

Globally, approximately 50 million individuals suffer from infections caused by filarial nematodes which lead to lymphatic filariasis. Moreover, an estimated 885 million people are at risk of contracting the disease worldwide [7]. 65% of those at risk of infection were from Southeast Asia [8]. Nepal has been identified as one of the 72 endemic countries for LF. 64 out of 77 districts in Nepal were identified as endemic, covering an initial at-risk population of 25 million [9]. As part of the global effort to eliminate the disease, Nepal conducted lymphatic filariasis mapping in 2001, 2005/2006, and then again in 2012 [9].

The study conducted between 2001 and 2002 identified that lymphatic filariasis is endemic in 33 out of 37 districts surveyed, with a prevalence ranging up to 40% in some areas. It highlights the high disease burden, particularly in regions with altitudes between 500–700 meters [10] In the Kapilbastu district, 9.9% of the population tested positive for microfilariae, with the highest prevalence among the elderly. [11]

The Global Program to Eliminate Lymphatic Filariasis (GPELF) was initiated by the World Health Organization (WHO) in the year 2000 with the aim to eradicate LF as a public health problem by the year 2020 [12,13]. Different strategies such as the Mass Drug Administration (MDA) and the provision of surgical treatment for hydrocele, basic hygiene, sanitation, and cleanliness measures for those with lymphoedema have been implemented to eliminate LF [4]. MDA programs have distributed over 9 billion antifilarial treatments in 72 endemic countries. 17 countries have achieved the goal of Eliminating Public Health Problems (EPHP) since 2000 [14].

In 2003, Nepal began MDA intervention to eliminate LF. The intervention continues today, and until 2021, a combination of Diethylcarbamazine (DEC) and Albendazole (ALB) was used. In 2022, Ivermectin was introduced in specific districts [9]

Baglung district exhibited a significantly lower MDA coverage of 75.69% in 2022 compared to other districts in Gandaki province. Previously Baglung also failed the Pre-TAS (Transmission Assessment Survey) and was waiting for TAS-I verification after completing the 14th round of MDA in 2023.

In MDA activities, health workers and drug distributors encountered some challenges such as a lack of medicine for adverse reactions and difficulty in distributing medicine to everyone in a single day [15]. Service providers play a crucial role in the success of MDA campaigns as they are responsible for distributing medication, educating communities about LF, addressing concerns, and ensuring compliance etc. These providers may encounter some challenges including issues with medicine supply, transportation, and financial matters [16].

Even though there has been a slight increase in mass drug administration (MDA) coverage, community acceptance still remains a significant challenge in its implementation. Understanding key factors such as knowledge, perceptions, and beliefs regarding lymphatic filariasis (LF) is critical to addressing this gap. Gaining these insights can help improve MDA strategies, maximize community acceptance, and contribute to the successful elimination of LF [17]. Therefore, the aim of this study is to assess the acceptability and implementation fidelity of mass drug administration for lymphatic filariasis in Baglung Municipality. Specifically, this study seeks to answer the research question: “What is the acceptability and implementation fidelity of mass drug administration for lymphatic filariasis in Baglung Municipality?”

## Methods and materials

### Setting and recruitment

The study was conducted in the community, targeting the administrative unit (wards) of Baglung Municipality. The study population were individuals aged ≥18 years who were eligible to participate in the MDA campaign based on the eligibility criteria.

Administratively, Baglung municipality was divided into 14 wards, and a simple random sampling method was applied for quantitative data collection in all 14 wards.

### Study design and sample size

A cross-sectional concurrent mixed method study was conducted. A total of 272 participants were included in this study, determined using Cochrane’s formula for a cross-sectional study [18]. All community members aged 18 years and older who were eligible for the MDA campaign and residing in Baglung municipality were included in the study.

For qualitative data, purposive sampling was used to ensure a diverse representation in terms of age, socio-economic status, location, and the urban/rural variations within the community. 3 focus group discussions (FGD) were conducted in the selected community of Baglung municipality in April 2024. Data collection was ceased as the repetition of information was seen and saturation was achieved. A total of (n = 25) were included for the FGD.

### Data collection

Data collection was conducted using paper-based questionnaires and was entered in EpiData version 3.1 For acceptability and other variables the structured questionnaires used in the previous research were used and modified as per necessity with extensive literature review and consultation with the experts [12,19,20]. A proportionate sampling method was applied to select households, followed by a lottery method to choose participants. Structured questionnaires, validated through pretesting and supervisor consultation were used, and direct interviews were conducted in Nepali in participants homes. All FGDs were performed at a location convenient to the participant’s homes or nearby places using guidelines developed in consultation with the expert. The key themes focused on by the FGD were knowledge of LF, perceptions of MDA, trust in health workers, concerns about side effects, and the influence of community norms. Data collection started on 27^th^ April 2024 and ended on 15^th^ May 2024.

#### Knowledge on LF and MDA.

For knowledge of LF six questions were included covering topics such as perceived modes of LF transmission, primary causes, belief in asymptomatic LF cases, personal concern regarding LF, beliefs about LF treatability, and preventability of LF. The knowledge regarding filariasis was categorized as poor and good. The score ranged from 0 to 6 and the cut-off point was obtained as 3.34 based on the mean score. Similarly, for knowledge on MDA, the questionnaire included inquiries about respondents awareness of the MDA campaign against LF, the sources through which they obtained information about the campaign, and their understanding of the purpose of the drug distribution exercise, etc. The knowledge regarding MDA was categorized as poor and good. The score ranged from 3 to 13 and the cut-off point was obtained as 8.84 based on the mean score. The correct responses were coded as “1” while incorrect and “do not know” responses were coded as “0”.

#### Attitude regarding LF, MDA, and service provider.

Attitudes towards LF, MDA, and service providers were assessed using a Likert scale. Participants were asked to indicate their level of agreement or disagreement with each statement, ranging from “Disagree a lot” to “Agree a lot.” For attitude towards LF, 7 statements covered perceptions of LF severity, personal risk, societal attitudes towards individuals with lymphoedema, and willingness to interact with affected individuals. For attitude towards MDA, 4 statements were presented, covering cultural acceptance of LF pills, personal feelings about taking LF pills, the perceived importance of MDA for community health, and individual willingness to take LF pills regardless of illness status. Similarly, towards service providers attitude, 6 statements covered trust in the drug distribution team, the extent of household visits by distributors, household awareness about drug distributions, perceptions of service providers’ attitudes and knowledge, and the adequacy of counseling provided before drug administration. The score for the attitude regarding lymphatic filariasis ranged from 12-35, likewise, for the attitude regarding MDA the score ranged from 7-20 and for the attitude regarding service provider score ranged from 17-30. The attitude regarding lymphatic filariasis, MDA and service provider was categorized as poor and good according to the threshold formula, and the cut-off point was obtained as 24.14, 15.32 and 23.54 respectively based on the mean score.

#### Coverage and compliance.

Participant’s coverage and compliance with MDA campaigns for LF were assessed through 8 questions. These questions addressed recall of past campaigns, receipt, and consumption of LF drugs, reasons for consumption or non-consumption, household coverage, means of verification, and past pill-taking history

#### Perceived fear of adverse effects and rumours regarding the MDA campaign.

Participant’s fear of adverse effects and rumours regarding the MDA campaign was assessed through 7 different questions. These questions addressed the concern and fear of adverse effects including the instances of adverse effects after consuming medicine. The questionnaires also looked at how aware people were of rumors about the MDA campaign, what the rumors were, and where they heard them, such as social media, community etc. It also studied how these rumors affected their decision to join the MDA campaign.

#### Acceptability of treatment for LF.

The acceptability of the treatment for Lymphatic Filariasis (LF) was assessed using a Likert scale consisting of 9 statements. Participants were asked to express their level of agreement, ranging from “Disagree a lot” to “Agree a lot,” regarding the effectiveness of the drugs against LF, itching, and intestinal worms, as well as their willingness to undergo the treatment again, recommend it to relatives, adjust their family’s routine for treatment, and overall satisfaction with the treatment’s effectiveness for community health problems. A composite acceptability score summed the values from nine indicators (range 9–36) with 22.5 as the threshold of acceptability [20,21]. This scale aimed to measure the community’s perception of the treatment’s acceptability and effectiveness in addressing LF-related health issues

### Data analysis

Data analysis was conducted using IBM SPSS version 22 software. Descriptive statistics, such as median, Inter interquartile range, and percentages, were employed to describe sociodemographic characteristics and levels of knowledge regarding LF, MDA, and other attributes. Chi-square tests were used to identify significant differences in acceptability and covariates such as socio-demographic variables including age, educational level, and household income, with a significance threshold set at p-values less than 0.05. Logistic regression was also run to identify the factors associated with the acceptability. Qualitative data from interviews were transcribed, translated into English, and thoroughly reviewed. Initial coding of transcripts was performed, which was refined through discussions with the expert, and then data triangulation was done to formulate the appropriate the theme.

### Ethical considerations

Data was collected after acquiring Ethical clearance from IRC, Pokhara University. The ethical approval was obtained from the Institutional Review Committee of Pokhara University with (reference number 131/2080/2081) on 26^th^ April 2024. Participants were fully informed regarding study objectives and the study was conducted only after the informed consent is signed by the participant. No direct benefit nor any risk was involved for the participants in this study. Confidentiality of the information was maintained and collected data were used only for the research purpose.

## Results

The study included 272 participants yielding a response rate of 100%, ensuring comprehensive representation across all retrieved interview schedules.

### Participants characteristics

As indicated in Table 1, the highest proportion of the participants were aged between 26–35 years, followed by 36–45 years (23.57%) with the median age being 39 years, with participants ranging from 19 to 78 years old, (Q_1_ ~ Q_3)_ = (30 ~ 50). Most of the participants were female (55.1%). Almost two-fifths of the participants were Brahmin followed by Dalits. Most of the participants were Hindu (95.2%). One-third of the participants had basic education. The majority 83.1% of the participants were Married. Agriculture and business were the major household occupations among the participants with 40.1% and 23.5% respectively. The average family size was found to be 4 and the median household income was found to be Rs. 30000 with IQR (Q_1_ ~ Q_3)_ = (20,000 ~ 50,000).

**Table 1:** Socio-demographic characteristics (Quantitative study).

Variables	Frequency (n = 272)	Percentage (%)
**Age in years**		
<26	45	16.5
26-35	71	26.1
36-45	64	23.5
46-55	48	17.6
≥56	44	16.2
Median = 39, (Q_1_ ~ Q_3)_ = (30 ~ 50) Min = 19, Max = 78		
**Gender**		
Male	122	44.9
Female	150	55.1
**Ethnicity** ^*^		
Brahmin/Chhetri	106	39.0
Dalit	82	30.1
Janajati	76	27.9
Religious minorities	8	2.9
**Religion**		
Hindu	259	95.2
Buddhist	7	2.6
Christian and Muslim	6	2.2
**Educational status**		
Illiterate	30	11.0
Informal	33	12.1
Basic	92	33.8
Secondary	74	27.2
Bachelors and Above	43	15.8
**Marital status**		
Married	226	83.1
Single	36	13.2
Widowed	10	3.7
**Household Employment**		
Agriculture	109	40.1
Business	64	23.5
Job	27	9.9
Foreign Employment	11	4.1
Daily wages	4	1.5
Unemployed	50	18.4
Retired	7	2.6
**Family Size**		
≤ 4 members	114	41.9
>4 members	158	58.1
Median = 5.0, (Q_1_ ~ Q_3)_ = (4 ~ 6) Min = 1, Max = 13		
**Household Income (In Nrs)**		
≤ 30000	148	54.4
> 30001	124	45.6
Median = 30000, (Q_1_ ~ Q_3)_ = (20,000 ~ 50,000) Min = 3000, Max = 200000		

* Brahmin/Chhetri: High Hindu Nepalese caste, Dalit: communities who, by virtue of atrocities of caste-based discrimination and untouchability.

Table 2 reflects the socio-demographic profile of participants included in the qualitative study. In contrast, all 25 participants in the Focus Group Discussions (FGDs) were community members.

**Table 2:** Participants characteristics (Qualitative study).

FGD’s	Male	Female	Total
FGD 1	0	7	7
FGD 2	2	8	10
FGD 3	4	4	8
	**Total**		**25**

### Coverage and compliance of the participants regarding MDA

Table 3 depicts the coverage and compliance of the MDA campaign among the participants. A total of 4% of participants could not recall the 14^th^ round of the MDA campaign. Among the total participants, 91.5% received and swallowed the medicine. The majority of the participants (62%) reported receiving a verification card after swallowing medicine. The majority 88.4% stated that health workers motivated and counseled them to take the drug against lymphatic filariasis. Additionally, 11.4% of participants noted that their family members did not receive the medicine during the campaign.

**Table 3:** Coverage and compliance.

Variables	Frequency	Percentage (%)
**Remember MDA campaign (n = 272)**		
Yes	261	96
No	11	4
**Received medicine against LF (n = 272)**		
Yes	249	91.5
No	23	8.5
**Swallow medicine against LF? (n = 249)**		
Yes	249	100
**Means of Verification**+ **(n = 249)**		
Card	153	62
Ink	68	27
Register	28	11
Motivation for consuming the drug*+ **(n = 249)**		
Counselling by health worker	220	88.4
Motivated by children	11	4.4
Counselling by relatives	8	3.2
Social Media Influence	15	6
**Other family members consumed medicine in last round of MDA (n = 272)**		
Yes	241	88.6
No	31	11.4
Reasons for not consuming medicine*$ **(n = 23)**		
Do not have the disease	13	46
Didn’t know about MDA	2	8
Not present at the community during the campaign	4	17
Drug distributors didn’t visit home	1	4
Not present at home during the visit	2	8
Not convinced about the need for the drugs	2	8
Fear of side effects	10	42
**No of times medicine consumed before (n = 272)**		
Never	15	5.5
Once	35	12.9
Twice or more	222	81.6

* Multiple response questions

+ Means of verification and motivation for consuming the drugs is only applicable for 249 participants who took the medicine

$ Reasons for not consuming the medicine is only applicable for 23 participants who didn’t take the drug

Among the non-compliant group (n = 23), 46% quoted their lack of illness as a reason for not taking the drug, while 42% expressed fear of potential side effects. Furthermore, when queried about the frequency of medication consumption during the 1^st^ to 14^th^ rounds of the MDA campaign, 81.6% emphasized having taken the drug more than twice, whereas 5.5% admitted to never having taken it.

*“Mostly, I went to the booth to consume the medicine, but sometimes when I couldn’t go, the FCHV brought it to my home, and we consumed it there. There are only two of us here now; my son and daughter are in Kathmandu and have always missed the campaign” (P1, FGD1)*

*“FCHV’s and other health workers came to my house to provide the medicine and they marked our house with chalk after consuming the medicine” (P4, FGD-1)*

*“I consumed the medicine at the booth established at health post itself and verification card was given to us after swallowing the medicine” (P7, FGD-1)*

*“Me and my husband were at home when health workers visited for MDA, but our children were at school at that time, I was worried that they didn’t get to consume the medicine. Later, I found out that my children also consumed the medicine at school” (P7, FGD-1)*

*“There are a lot of people who have not swallowed the medicine. I heard they throw it away after taking it. They would say that they have consumed it, but they just throw it away” (P5, FGD-2)*

*“MDA campaign has been going on for a while now, most people have taken the medicine for only 5-6 years. I have taken it for the complete 14 years, whereas some people are still missed” (P3, FGD-3)*

**Box 1**

Participants shared varied experiences regarding their participation in the MDA campaign. Some took the medicine at booths while some consumed it after abvailability at home by FCHVs and health workers. Verification methods included a card system with 62% receiving the card, inking nails, and marking houses. Despite these efforts, some discarded the medicine without consuming it. Participation varied, with some taking the medicine once and others participating for several years. Consistent participation was reported by some over 14 years, although some were still missed.

### Knowledge and attitude on LF and MDA

Table 4 represents the knowledge and attitude on LF and MDA among the participants. The knowledge regarding LF and MDA was characterized as poor and good while attitudes regarding LF, MDA and service providers were characterized as positive and negative according to the threshold formula. The cut-off point was obtained as 3.34, 8.84 for knowledge and 24.14, 15.32. and 23.54. for attitude. The findings demonstrate that 48.5% of participants had good knowledge for LF, similarly 84.2%, demonstrated a good knowledge on MDA. Whereas only 45.2% showed positive knowledge on LF, similarly, 59.3% of participants showed a positive attitude towards MDA. 64.3% of participants expressed a positive attitude towards the service providers.

**Table 4:** Knowledge and attitude regarding LF and MDA.

Variables	Frequency (n = 272)	Percentage (%)
**Knowledge regarding filariasis**		
Poor knowledge	140	51.5
Good knowledge	132	48.5
**Knowledge regarding MDA**		
Poor knowledge	43	15.8
Good knowledge	229	84.2
**Attitude regarding filariasis**		
Negative Attitude	149	54.8
Positive Attitude	123	45.2
**Attitude regarding MDA campaign**		
Negative Attitude	111	40.8
Positive Attitude	161	59.3
**Attitude regarding MDA service provider**		
Negative Attitude	97	35.7
Positive Attitude	175	64.3

### Knowledge regarding LF and MDA

*“If the mosquito bites the person who has received the injection against it then because of that it gets transmitted” (P1, FGD-1)*

*“Filariasis is something in which someone has a swollen leg and gains weight, it happens in someone’s leg, someone’s hand, neck, or other part of the body” (P1, FGD-2)*

*“I heard about MDA from the also from the FCHV and news as well. My child also got to learn about it from school and it works against the worm” (P3, FGD-1)*

*“Yes, there was a medicine administration program against filariasis, but no one was voluntarily willing to take the medicine” (P1, FGD-3)*

**Box 2**

**Knowledge**: Participants exhibited varying levels of knowledge regarding lymphatic filariasis and MDA. Some believed it was transmitted through mosquito bites, particularly from female mosquitoes or those that bite individuals who have received an injection. Others associated the disease, known locally as Hattipaile, with symptoms like swollen limbs or weight gain, and thought it could affect various parts of the body. Regarding knowledge of MDA, sources of information included FCHVs, health workers, news, and social media. However, despite awareness, there was also a perception that voluntary participation in the MDA program was low.

### Attitude towards LF, MDA, and service providers

*“I do not have any feelings of hatred towards people with filariasis. If we discriminate against that person then it is called violence” (P9, FGD-2)*

*“Some people have been taking this as a conspiracy of the government as every year they come to feed us the medicine but i know that we should consume this medicine to prevent the transmission of disease” (P7, FGD 2)*

*“Previously medicines were not provided to chronically ill patients but now healthcare providers themselves visited and provided them the medicine. Previously, some people did not consume, and because of this, the disease was seen, so health workers counseled everyone to consume the medicine. I am thankful towards them” (P10, FGD-2)*

*“Health workers counseled us properly before providing the medicine. They informed us what the medicine was for. Otherwise, how to consume it without knowing? I am grateful towards them” (P9, FGD-2)*

*“I have a fear towards lymphatic filariasis. The picture of a patient with lymphatic filariasis is quite disgusting, and after knowing about medicine for the same disease, it was fearful” (P5, FGD-3)*

**Box 3**

**Attitude** Participants attitudes towards lymphatic filariasis differed. Some felt compassion and believed that discriminating against those with the disease was wrong. Others expressed fear, finding images of the disease disturbing and feeling afraid despite knowing there was medication available. Some participant showed their doubt about the effectiveness of mass drug administration, suggesting people may discard the medicine, indicating a lack of trust. Another acknowledges its importance in disease prevention, despite suspicions about government motives, showing belief in its benefits despite concerns. Participants expressed gratitude towards MDA service providers for their informative counseling and provision of medicine. They appreciate being properly informed about the purpose of the medicine, highlighting the importance of understanding its use.

### Fear of side effects/adverse effects among participants

Table 5 depicts the fear among participants regarding the potential side effects of the medicine. More than two-fifths (43%) worried about these side effects, while 37.1% reported actual fear. Additionally, only 17% of participants experienced minor side effects after swallowing the medicine.

**Table 5:** Fear of side effects/adverse effects.

Variable	Frequency	Percentage (%)
**Concerned about the potential side effects (n = 272)**		
Yes	118	43.0
No	154	57.0
**Fearful about the side effects (n = 272)**		
Yes	101	37.1
No	171	62.9
**Had side effects after consuming medicine (n = 249)**		
Yes	42	17.0
No	207	83.0

*“Yes, I am worried about the side effects. I feel I might be affected after consuming the medicine” (P6, FGD-1)*

*“Yes, after consuming the medicine I had nausea, I felt weak and couldn’t work properly for 2 days” (P8, FGD-2)*

*“Yes, after consuming the medicine I had mild nausea. I immediately informed the health worker about it. After getting some rest I recovered myself” (P7, FGD-3)*

*“I didn’t notice any such things in me and my family but yes, some people in my community said that they had a fever after consuming the medicine” (P8, FGD-3)*

**Box 4**

Participants reported mixed experiences with side effects after consuming the medicine. Some experienced nausea, vomiting, or fever, while many reported no adverse effects. One participant felt weak and unable to work for two days, and another recovered after resting. Despite these side effects, not everyone reported negative reactions.

### Rumours regarding MDA among participants

Table 6 depicts that most of the participants (52.9%) were exposed to various rumours surrounding the MDA campaign. Among these rumours, the majority (94%) reported hearing about potential severe side effects associated with consuming the medication. Interestingly, participants identified friends (67%) as the primary source of these rumours, Despite the prevalence of rumours, only 13.2% of respondents acknowledged that these rumours had influenced their decision to participate in the MDA campaign.

**Table 6:** Rumours regarding MDA among participants.

Variable	Frequency	Percentage (%)
**Have heard about rumours (n = 272)**		
Yes	144	52.9
No	128	47.1
Types of rumours* **(n = 144)**		
The drugs cause severe side effects	135	94.0
The drugs are not effective against LF	6	4.0
MDA campaign is a government conspiracy	5	3.0
People died after consuming medicine	1	1.0
Source of Rumours* **(n = 144)**		
Social Media	19	13.0
Friends	96	67.0
Community people	53	37.0
**Rumours or misinformation affected from taking part in MDA? (n = 144)**		
Yes	19	13.2
No	125	86.8

* Multiple response questions

*“Someone in my community said that, consuming this many medicines might affect us, we might feel dizzy, or we might even die” (P8, FGD-2)*

*“I have heard that there are bacteria in the saltwater of the sea; if you drink that water, it will be transmitted” (P2, FGD-3)*

*“No, I have not heard about any such rumours regarding MDA. But yes, we were told minor side effects can be seen because of medicine” (P4, FGD-3)*

**Box 5**

Participants had varied exposure to rumours about MDA. Some heard that taking multiple medicines could cause dizziness or even death. Others were not aware of such rumours but were informed about potential minor side effects by the health workers.

### Acceptability of mass drug administration among participants

The acceptability was categorized as acceptable and not acceptable according to the threshold formula and the cut-off point was obtained as 22.5. The Table 7 demonstrates the distribution of participant’s acceptability toward the MDA campaign. Four out of every five participants accepted the MDA campaign.

**Table 7:** Acceptability of MDA campaign.

Variable	Frequency (n = 272)	Percentage (%)
**Acceptability of MDA Campaign**		
Acceptable	219	80.5
Not Acceptable	53	19.5

### Association between characteristics/variables and Acceptability

The data presented in Table 8 demonstrates the association between socio-demographic factors and the acceptance of the MDA. There were no statistically significant variations in acceptance of MDA according to their age, gender, ethnicity, religion, marital status, family size, or income level (all p > 0.05). Conversely, a significant difference was observed between educational status and acceptance (p = 0.012). Participants with higher education had more acceptance than those who were less educated. While occupation did not show significant differences (p = 0.383), a slightly larger proportion of individuals involved in agriculture (77.1%) found the program acceptable compared to those in other professions.

**Table 8:** Association between characteristics/variables with Acceptability.

Variables	Categories	Acceptability		Chi-sq	p-value
		Not Acceptable n (%) 53 (19.5)	Acceptable n (%) 219 (80.5)		
**Age (In Yrs.)**	≤40	23 (15.3)	127 (87.4)	3.675	0.055
	>40	30 (24.6)	92 (75.4)		
**Gender**	Male	27 (22.1)	95 (77.9)	0.987	0.320
	Female	26 (17.3)	124 (82.7)		
**Ethnicity**	Advantaged Group	16 (15.1)	90 (84.9)	2.135	0.344
	Relatively advantaged	17 (22.4)	59(77.6)		
	Disadvantaged	20 (22.2)	70 (77.8)		
**Religion**	Hindu	53 (20.5)	206 (79.5)		0.079^+^
	Non-Hindu	0	13 (100)		
**Education**	Illiterate	11 (36.7)	19 (63.3)	6.345	**0.012** ^*^
	Literate	42 (17.4)	200 (82.6)		
**Marital status**	Married	43 (19.0)	183 (81.0)	0.179	0.672
	Others	10 (21.7)	36 (78.3)		
**Occupation**	Agriculture	25 (22.9)	84 (77.1)	3.058	0.383
	Business	8 (12.5)	56 (87.5)		
	Others	9 (18.4)	40 (81.6)		
	Unemployed	11 (22.0)	39 (78.0)		
**Family size**	≤4 members	21 (18.4)	93 (81.6)	0.142	0.707
	>4 members	32 (20.3)	126 (79.7)		
**Income (In Nrs.)**	≤30000	30 (19.1)	127 (80.9)	0.034	0.854
	>30001	23 (20.0)	92 (80.0)		
**Knowledge on filariasis**	Poor knowledge	30 (21.4)	110 (78.65)	0.694	0.405
	Good knowledge	23 (17.4)	109 (82.6)		
**Knowledge on MDA**	Poor knowledge	12 (27.9)	31 (72.1)	2.309	0.129
	Good knowledge	41 (17.9)	188 (82.1)		
**Attitude regarding filariasis**	Negative attitude	34 (22.8)	115 (77.2)	2.334	0.127
	Positive attitude	19 (15.4)	104 (84.6)		
**Attitude regarding MDA**	Negative attitude	32 (28.8)	79 (71.2)	10.435	**<0.001** ^*^
	Positive attitude	21 (13.0)	140 (87.0)		
**Attitude regarding service providers**	Negative attitude	22 (22.7)	5 (77.3.0)	0.981	0.322
	Positive attitude	31 (17.7)	144 (82.3)		
**Concerned about potential side effects**	No	27 (17.5)	127 (82.5)	0.863	0.353
	Yes	26 (22.0)	92 (78.0)		
**Fearful of side effects**	No	23 (13.5)	148 (86.5)	10.691	**<0.001** ^*^
	Yes	30 (29.7)	71 (70.3)		
**Received Drugs against LF**	No	12 (52.2)	11 (47.8)	17.112	**<0.001** ^*^
	Yes	41 (16.5)	208 (83.5)		
**Heard about rumours regarding MDA**	No	20 (15.6)	108 (84.4)	2.297	0.130
	Yes	33 (22.9)	111 (77.1)		

+ Fisher exact value for cell frequency less than 5 was taken

* Statistically significant at p < 0.05

Table 8 also presents the association between knowledge-related factors and the acceptability of MDA against filariasis. No statistically significant association was observed between knowledge of filariasis and acceptability of MDA (p = 0.405). Similarly, no significant association was found between knowledge of MDA and acceptability (p = 0.129). Similarly, a statistically significant association was observed between attitude toward MDA and acceptability (p = 0.001), indicating that individuals with a good attitude toward MDA were more likely to find it acceptable compared to those with a poor attitude. However, no significant associations were found between attitude toward filariasis (p = 0.127) or attitude toward service providers (p = 0.322) and acceptability of MDA.

### Multivariate analysis of factors associated with Acceptability of Mass Drug Administration

Logistic regression was performed to find out the factors associated with the acceptability of MDA. The variables that were used in bivariate analysis were run on multivariate analysis as well, which were: age, gender, ethnicity, education, marital status, occupation, family size, income, knowledge regarding filariasis, knowledge regarding MDA, attitude regarding filariasis, attitude regarding MDA, attitude regarding service providers, concerned about potential side effects, fearful of side effects, drugs received against LF, heard rumors against LF. Table 9 presents the information regarding factors associated with the acceptability of MDA. For each variables, the table shows the unadjusted and adjusted odds ratio (OR), 95% confidence interval (CI), and p-value. The adjusted ORs were calculated after controlling for the effects of other variables in the final model.

**Table 9:** Multivariate analysis of factors associated with acceptability of mass drug administration.

Variables	Unadjusted OR (95% CI)	p-value	Adjusted OR (95% CI)	p-value
**Age**				
≤40	Ref		Ref	
>40	0.555 (0.303-1.018)	0.057	0.658(0.312-1.389)	0.272
**Gender**				
Male	Ref		Ref	
Female	1.355 (0.743-2.473)	0.321	1.634 (0.756-3.533)	0.212
**Ethnicity**				
Advantaged Group	Ref		Ref	
Relatively advantaged	0.617 (0.289-1.316)	0.201	0.701 (0.299-1.645)	0.414
Disadvantaged	0.622 (0.301-1.288)	0.982	0.721 (0.313-1.663)	0.443
**Education**				
Illiterate	Ref		Ref	
Literate	2.757 (1.222-6.220)	**0.015** ^*^	2.264 (0.849-6.034)	0.102
**Marital Status**				
Married	Ref		Ref	
Unmarried	0.846 (0.390-1.837)	0.672	0.948 (0.351-2.564)	0.917
**Occupation**				
Agriculture	Ref		Ref	
Business	2.803 (0.877-4.948)	0.096	1.828 (0.628-5.318)	0.268
Others	1.323 (0.565-3.094)	0.519	1.449 (0.509-4.122)	0.487
Unemployed	1.055 (0.472-2.359)	0.896	0.846 (0.289-2.479)	0.760
**Family size**				
≤4 members	Ref		Ref	
>4 members	0.889 (0.482-1.640)	0.707	1.005 (0.489-2.067)	0.988
**Income (In Nrs.)**				
≤30000	Ref		Ref	
>30001	0.945 (0.516-1.732)	0.854	0.998 (0.469-2.123)	0.996
**Knowledge regarding Filariasis**				
Poor knowledge	Ref		Ref	
Good knowledge	1.292 (0.706-2.365)	0.405	0.698 (0.324-1.463)	0.332
**Knowledge regarding MDA**				
Poor knowledge	Ref		Ref	
Good knowledge	1.775 (0.841-3.747)	0.132	2.418 (0.991-5.895)	0.502
**Attitude regarding Filariasis**				
Negative attitude	Ref		Ref	
Positive attitude	1.618 (0.870-3.011)	0.129	1.305 (0.631-2.699)	0.473
**Attitude regarding MDA**				
Negative attitude	Ref		Ref	
Positive attitude	2.700 (1.459-4.998)	**0.002** ^*^	2.109 (1.068-4.166)	**0.032** ^*^
**Attitude regarding the service provider**				
Negative attitude	Ref		Ref	
Positive attitude	1.363 (0.738-2.517)	0.323	0.941 (0.425-2.089)	0.884
**Concerned about potential side effect**				
No	Ref		Ref	
Yes	0.752 (0.412-1.373)	0.354	1.682 (0.695-4.071)	0.249
**Fearful of side effects**				
Yes	Ref		Ref	
No	2.717 (1.474-5.016)	**<0.001** ^*^	2.895 (1.181-7.092)	**0.020** ^*^
**Drugs received against LF**				
No	Ref		Ref	
Yes	5.535 (2.286-13.397)	**<0.001** ^*^	3.375 (1.156-12.075)	**0.028** ^*^
**Heard about rumours regarding LF**				
No	Ref		Ref	
Yes	0.623 (0.337-1.153)	0.132	1.243 (0.564-2.738)	0.589

* Statistically significant at p < 0.05

For the education, the unadjusted OR for literate individuals was 2.757 (95% CI: 1.222-6.220, p = 0.015), indicating that literate individuals were 2.757 times more likely to accept MDA compared to illiterate individuals. This suggests a significant association between literacy and MDA acceptability in the unadjusted analysis. However, after adjusting for other variables, the adjusted OR for literate individuals was 2.264 (95% CI: 0.849-6.034, p = 0.102), which is no longer statistically significant. This implies that while there initially appeared to be a significant association between literacy and MDA acceptability, this association diminished when accounting for other factors, indicating that literacy alone may not be a significant predictor of MDA acceptability once other variables are considered.

The unadjusted odds ratio (OR) for individuals with a good attitude towards MDA was 2.700 (95% CI: 1.459-4.998, p = 0.002), indicating that these individuals were 2.7 times more likely to accept MDA compared to those with a poor attitude. This association remained significant after adjusting for other variables, with an adjusted OR of 2.109 (95% CI: 1.068-4.166, p = 0.032), suggesting that a positive attitude towards MDA significantly increases its acceptability. Individuals who were fearful of side effects had an unadjusted OR of 0.368 (95% CI: 0.199-0.679, p = 0.001), indicating they were less likely to accept MDA. This association remained significant after adjustment, with an adjusted OR of 0.345 (95% CI: 0.141-0.846, p = 0.020), confirming that fear of side effects is a strong deterrent to accepting MDA. The unadjusted OR for receiving drugs against LF was 5.535 (95% CI: 2.286-13.397, p = 0.001), indicating those who received drugs were significantly more likely to accept MDA. This association remained significant after adjustment, with an adjusted OR of 3.375 (95% CI: 1.243-9.168, p = 0.028), suggesting that receiving drugs against LF increases the likelihood of MDA acceptability.

### Discussion

This study aimed to assess the acceptability of mass drug administration for lymphatic filariasis in Baglung Municipality. The findings of this study reveal majority of participants had an acceptance for the MDA campaign in Baglung Municipality, with four-fifths of participants considering it acceptable.

Less than half of the participants had good knowledge regarding LF. This aligns with the study conducted in southern Ghana where 45.8% of participants reported receiving information about LF from healthcare providers and MDA programs. However, only a small fraction (6.9%) understood that LF is transmitted by mosquitoes, indicating a significant gap in knowledge despite some level of information dissemination [22]. Similarly, a study in Nigeria involving 243 participants found that only 35% had heard of LF, and only one-fourth had good knowledge of the disease. This could be because of several factors. First, the quality and clarity of the information provided may not be adequate. Healthcare providers and MDA programs might be distributing information, but if it is not communicated effectively or if it is too complex, it may not be understood by the community members. Additionally, cultural beliefs, misinformation, and rumours can also hinder the effective transmission of knowledge. For instance, misconceptions about the disease’s transmission or the safety of the medication can overshadow factual information. It is crucial to evaluate not just the dissemination of information, but also how it is received and understood by the target audience.

In contrast, another study conducted in Ghana reported high awareness of LF among both treated and non-treated groups, with awareness percentages reaching 96.9% and 98.6%, respectively. This study identified personal connections within the community and media sources as significant channels for disseminating information about LF [23]. This suggests that local networks and media could be an effective strategy to enhance knowledge levels in Baglung as well. Overall, the comparison with other studies underscores the variability in knowledge levels across different regions and the critical role of customized information dissemination strategies.

In one study in Philippines, 89.1% of the sampled population reported being aware of MDA, and nearly all believed it was intended for LF. This high level of awareness is comparable to Baglung, where almost similar proportion demonstrated good knowledge. In Baglung, the Majority, 84.2%, demonstrated a better understanding, categorized as good knowledge. It also supports the idea that well-implemented awareness programs as well as MDA programs can significantly enhance public knowledge and acceptance of MDA [24]. Also, this alignment could be attributed to similar strategies employed in both contexts, such as extensive national campaigns that effectively disseminate information. In Baglung, the Majority, 84.2%, demonstrated a better understanding, categorized as good knowledge contrasting sharply with a study conducted in Kerala, where a majority (67.4%) were unfamiliar with the term MDA. This suggests that the MDA campaigns in Baglung were more effective or that the population were more pervasice and educated [25].

Furthermore, this study showed varied attitudes towards lymphatic filariasis (LF), mass drug administration (MDA), and service providers. A positive attitude toward LF in this context does not reflect a high level of knowledge about the disease or acceptance of MDA, but rather refers to an empathetic non-stigmatizing view towards people with LF, reduced social prejudice, and recognition of the seriousness of the disease. Conversely, a negative attitude suggests social stigma, avoidance behavior, or a minimization of LF as a public health concern. Specifically, 45.2% of participants had a positive attitude toward LF, while 59.3% and 64.3% had good attitudes toward the MDA campaign and service providers, respectively. The positive attitude towards the MDA campaign significantly associated with its acceptability (p = 0.001), whereas attitudes towards LF and service providers did not show a significant association. These findings align with the study conducted in Philippines that highlights the importance of favorable attitudes toward MDA in enhancing program uptake. This study found that positive perceptions and trust in health workers were crucial for MDA acceptance [24]. However, contrary evidence from other studies suggests that negative attitudes toward LF and mistrust in healthcare providers can significantly impede public health initiatives, such as in a study in Indonesia, which showed a strong correlation between trust in healthcare services and MDA participation rates [26]. The finding from this study revealed that, health worker counseling was a primary motivator for MDA consumption, with 88.4% of individuals consuming the medicine due to health worker engagement which can be compared with another similar study done in Dhading, Kapilvastu and Kailali which highlighted that those visited by health workers during the MDA campaign had significantly higher compliance (75.9% P < 0.001). This underscores the importance of direct interaction and personalized counseling in enhancing MDA compliance [27]. This alignment could be attributed to similar strategies employed in both contexts, such as extensive national campaigns that effectively disseminate information. The success of these campaigns often involves direct engagement with the community, including home visits by health workers to provide medicine and inform residents about the purpose and importance of MDA. This personalized approach ensures that the information is not only distributed but also understood, thereby enhancing overall awareness and knowledge about MDA.

This study found a significant association between attitudes toward MDA and its acceptability (p = 0.001). Specifically, 59.3% of participants with a positive attitude were more likely to accept MDA compared to 40.8% with a negative attitude. This indicates that individuals with a good attitude toward MDA are more likely to accept and comply with the program. Positive attitudes likely result from understanding the benefits, trust in healthcare providers, and positive past experiences. Therefore, enhancing public attitudes through education, clear information, and community engagement is crucial for improving MDA uptake and achieving its target.

The findings of this study reveal a high level of acceptability for the MDA campaign in Baglung Municipality, with 80.5% of participants considering it acceptable. This finding aligns well with the prior study conducted in Guyana across four different regions which showed that the mean acceptability scores ranged from 24.6 to 29.3, surpassing the threshold of 22.5, indicating a generally high level of acceptability [20]. The findings from this study also comes in line with another multicenter, community-based, mixed-method study conducted in five different countries, Fiji, Haiti, India, Indonesia and PapuaNewGuinea. The multicenter study emphasizes the acceptability of specific drug regimens across various countries, with mean scores ranging from 26.8 to 33.7 showing the high acceptability [21]. Both studies reported high levels of acceptability for the MDA campaigns. Both studies underscore the importance of effective communication and awareness about the MDA program, highlighting that knowledge and perception of drug safety are critical to community acceptance. While regional variations exist, the consistent high acceptability across different contexts suggests that tailored, localized strategies and sustained education efforts are essential for the success of MDA initiatives Despite the differences in scope and methodology, both studies underscore the importance of professional treatment delivery and community engagement in supporting acceptance.

This study indicates a high level of community participation in the MDA program, with 96% recalling the campaign and 91.5% receiving the medication. 100% of the individuals who received the medicine have consumed it, primarily motivated by health worker counseling (88.4%). These results align closely with a study reporting a 95.5% coverage and 71.6% compliance in a similar study in Nepal, underscoring the effectiveness of health worker involvement as well [28].

The findings from this study revealed that fear of side effects and rumours about MDA significantly influence participants perceptions and acceptance of the program. This aligns with existing literature, which identifies fear of adverse effects as a major barrier to MDA participation.. For instance, a study conducted in Nepal [28] found that a significant proportion of participants identified fear of side effects as a major concern affecting their willingness to participate in MDA programs. The study further indicated that individuals who had prior knowledge of potential side effects were less likely to be non-compliant, with non-compliance rates being notably lower among those informed about side effects (9.4% vs. 33.2%, P < 0.001) In Baglung, 43% of participants were concerned about potential side effects, and 17% experienced minor side effects post-consumption. This fear significantly influenced their acceptance of the MDA program which is also consistent with findings from similar studies in Sri Lanka [29]. The significant influence of fear of side effects and rumours on MDA acceptance likely occurs due to several factors. Lack of comprehensive and clear information about the potential side effects of the medication can lead to heightened anxiety and fear among community members. When people are not fully informed or misunderstand the nature and likelihood of side effects, they may be more susceptible to believing and spreading rumours

Moreover, 52.9% of participants reported hearing rumours about MDA, predominantly about severe side effects, primarily spread by friends in this study which mirrors findings by [25]. and (Krentel, Fischer, and Weil, 2013). which noted that misinformation, often spread through social networks, significantly hindered MDA uptake in various regions. However, only 13.2% of our participants reported that rumours affected their participation, suggesting that effective health communication can mitigate the impact of such misinformation. This is consistent with another study in India which shows that strong community health education and trust in health workers could mitigate the negative effects of rumours on participation rates [30].

This study also showed a significant association between fear of side effects and lower MDA acceptability (p = 0.001), highlighting the importance of addressing these fears to improve program uptake. Equally, while rumours were associated with a slight decrease in acceptability, this relationship was not statistically significant (p = 0.130). These results underscore the need for comprehensive strategies that include clear communication about side effects and efforts to counteract rumours, as supported by a study from India [16].

### Limitations of the study

Our study’s reliance on self-reported data introduces potential biases, such as recall bias, which could impact the accuracy of participants reported knowledge, attitudes, and behaviors regarding MDA and LF. Generalizability of the findings limited to other areas of Nepal due to it’s specific geographical, cultural and socio economic context of the participants. Furthermore, the qualitative method was limited with limiting the engagement of other stakeholders too. The interpretation of this data is subjective and may be influenced by the researchers perspectives and might lead to biases.

## Conclusion

This study on the acceptability of the Mass Drug Administration (MDA) program for lymphatic filariasis (LF) in Baglung Municipality has described the factors influencing the program’s acceptability and success. Community awareness and participation regarding MDA were good, but the program still faced challenges, including fears of side effects, persistent rumours, and varying levels of knowledge about LF.

This study found that, despite the limited knowledge of LF among participants, there was still a high level of participation in the MDA campaign. Similarly, Baglung Municipality showed a high acceptability of MDA among the community, with the majority of participants rating the campaign positively. The success in acceptability was largely due to a high degree of trust in healthcare providers, which somehow helped to mitigate concerns about side effects and counter rumours. Despite limited knowledge about LF, transparent and proactive communication by health workers including clear messaging played a crucial role in gaining community support and participation.

## Supporting information

S1 Data XXX.
(SAV)
